# Nutrient requirements and growth physiology of the photoheterotrophic Acidobacterium, *Chloracidobacterium thermophilum*

**DOI:** 10.3389/fmicb.2015.00226

**Published:** 2015-03-27

**Authors:** Marcus Tank, Donald A. Bryant

**Affiliations:** ^1^Department of Biochemistry and Molecular Biology, Eberly College of Science, The Pennsylvania State UniversityPA, USA; ^2^Department of Chemistry and Biochemistry, Montana State UniversityBozeman, MT, USA

**Keywords:** bacteriochlorophyll, anoxygenic photosynthesis, photoheterotroph, thermophile, Acidobacteria

## Abstract

A novel thermophilic, microaerophilic, anoxygenic, and chlorophototrophic member of the phylum *Acidobacteria*, *Chloracidobacterium thermophilum* strain B^T^, was isolated from a cyanobacterial enrichment culture derived from microbial mats associated with Octopus Spring, Yellowstone National Park, Wyoming. *C. thermophilum* is strictly dependent on light and oxygen and grows optimally as a photoheterotroph at irradiance values between 20 and 50 μmol photons m^-2^ s^-1^. *C. thermophilum* is unable to synthesize branched-chain amino acids (AAs), l-lysine, and vitamin B_12_, which are required for growth. Although the organism lacks genes for autotrophic carbon fixation, bicarbonate is also required. Mixtures of other AAs and 2-oxoglutarate stimulate growth. As suggested from genomic sequence data, *C. thermophilum* requires a reduced sulfur source such as thioglycolate, cysteine, methionine, or thiosulfate. The organism can be grown in a defined medium at 51^∘^C (T_opt_; range 44–58^∘^C) in the pH range 5.5–9.5 (pH_opt_ = ∼7.0). Using the defined growth medium and optimal conditions, it was possible to isolate new *C. thermophilum* strains directly from samples of hot spring mats in Yellowstone National Park, Wyoming. The new isolates differ from the type strain with respect to pigment composition, morphology in liquid culture, and temperature adaptation.

## Introduction

“*Candidatus* Chloracidobacterium thermophilum” was first detected by bioinformatics analyses of metagenomic sequence data for phototrophic microbial mats from alkaline (∼pH 8.2) siliceous hot springs (50–65^∘^C) in Yellowstone National Park, Wyoming, USA (Bryant et al., 2007). Inferences from these analyses indicated that this previously uncharacterized bacterium had a photosynthetic apparatus that was highly similar to that of obligately anaerobic, anoxygenic, and chlorophotrophic members of the *Chlorobiales* (i.e., green sulfur bacteria). It was inferred that “*Ca*. C. thermophilum” synthesized BChl *a* and *c,* and that it had a photosynthetic apparatus comprising chlorosomes as light-harvesting antenna complexes, the Fenna-Matthews-Olson, BChl *a*-binding protein (FmoA), and homodimeric type-1 photochemical reaction centers. However, in contrast to green sulfur bacteria, BChl biosynthesis and some other cellular processes (e.g., tyrosine biosynthesis) apparently are oxygen-dependent (Bryant et al., 2007; Garcia Costas et al., 2012a).

Phylogenetic analyses of the sequences of 16S rRNA and RecA assigned the new bacterium to subdivision 4 of the highly diverse phylum, *Acidobacteria* (Barns et al., 1999; Bryant et al., 2007; Tank and Bryant, 2015). *C. thermophilum* is currently the only cultivated, phototrophic member of this phylum, and together with *Pyrinomonas methylaliphatogenes, Blastocatella fastidiosa,* and *Aridibacter famidurans* and *A. kavangonen*sis, *C. thermophilum* (Tank and Bryant, 2015) is one of the few axenic strains in this subdivision (Foesel et al., 2013; Crowe et al., 2014; Huber et al., 2014). At the time of its initial discovery, “*Ca*. C. thermophilum” extended the number of bacterial phyla known to have members capable of Chl -dependent phototrophic growth (i.e., chlorophototrophy) from five to six. These include the phyla *Cyanobacteria, Chloroflexi, Chlorobi, Proteobacteria*, *Firmicutes,* and now *Acidobacteria* (Bryant et al., 2007). This number has very recently expanded from six to seven because of the discovery of a BChl *a*-producing, anoxygenic photoheterotroph from the phylum *Gemmatimonadetes* (Zeng et al., 2014).

As opposed to the “*in silico*” evidence describing this organism, the existence of *C. thermophilum* was confirmed by identifying living cells that exhibited similar DNA signatures to those of the organism described by metagenomic analysis. Those cells were detected in a cyanobacterial enrichment generated from Octopus Spring and were used to initiate the characterization of *C. thermophilum*. The cyanobacterium in the enrichment, a *Synechococcus* sp., was rather easily eliminated by providing a mixture of carbon sources to the enrichment culture and by adding atrazine to inhibit the growth of the cyanobacterium (Bryant et al., 2007). However, in spite of considerable effort, attempts to eliminate two heterotrophic contaminants, *Anoxybacillus* sp. and *Meiothermus* sp., were unsuccessful over a period of years. This suggested that these bacteria were providing essential nutrients to *C. thermophilum*, removing growth inhibitory substances, and/or otherwise changing the culture conditions in a way that was essential for the growth of *C. thermophilum*.

Using physical methods (e.g., low-speed centrifugation) to obtain enriched populations of cells, it was possible to obtain highly enriched DNA preparations for *C. thermophilum*. This allowed the complete genome of the organism to be determined (Garcia Costas et al., 2012a). The genome sequence verified many of the inferences from the metagenomic data concerning this bacterium, and these data further revealed important clues about the physiology and metabolism of *C. thermophilum.* Consistent with inferences from the metagenomic data, the genome lacked genes for known CO_2_ fixation pathways, and it additionally lacked genes for oxidoreductases that could provide electrons for CO_2_ reduction. Moreover, the genome lacked all genes except those encoding aminotransferases for the synthesis of the branched-chain AAs, l-leucine, l-isoleucine, and l-valine. Surprisingly, genes encoding enzymes for the complete degradation of branched-chain AAs were present, however, (Garcia Costas et al., 2012a). The genome additionally lacked genes for nitrate reductase, nitrite reductase, nitrogenase, and assimilatory sulfate reduction. Based upon these findings, Garcia Costas et al. (2012a) concluded that *C. thermophilum* was an aerobic anoxygenic photoheterotroph. A metatranscriptomic study of the microbial mats from which the organism was enriched over a complete diel cycle suggested that *C. thermophilum* might require alternating oxic and anoxic conditions for optimal growth or might prefer constantly microoxic conditions (Liu et al., 2011, 2012).

Armed with all of this background information, we describe here how the insights gained from metagenomic, genomic, and metatranscriptomic studies were evaluated and used, in combination with biochemical and classical microbiological methods, to isolate an axenic culture of *C. thermophilum*. By establishing the conditions for axenic growth, we elucidated preferred carbon, nitrogen, and sulfur sources, optimum pH, temperature and light intensities as well as the specific oxygen relationship of *C. thermophilum*. Our findings have allowed us to isolate new axenic cultures of *C. thermophilum* strains directly from microbial mats in Yellowstone National Park.

## Materials and Methods

### Source Material

The original source material of the organism described here was cyanobacterial enrichment culture B^′^-NACy10o, which was derived from a sample collected by Allewalt et al. (2006) on July 10, 2002 from Octopus Spring at a site temperature of 51–61^∘^C. The genome sequence of the cyanobacterium in this enrichment culture, denoted as *Synechococcus* sp. JA-2-3B^′^ a(2-13), was reported by Bhaya et al. (2007). The enrichment culture was first simplified by elimination of the *Synechococcus* sp., which produced a stable co-culture of *C. thermophilum*, *Anoxybacillus* sp. and *Meiothermus* sp. as previously described (Bryant et al., 2007; Garcia Costas et al., 2012a). Procedures used to obtain the axenic type strain (strain B^T^; Tank and Bryant, 2015) are described here.

### Medium and Medium Preparation

In order to isolate an axenic culture of *C. thermophilum*, it was necessary to modify the medium several times in an iterative fashion. The *C. thermophilum* Midnight Medium (CTM medium) description that follows was the result of this process and was the basis for all further growth experiments. As described in the text, this medium was modified in some experiments to test various parameters. Several stock solutions were used during the preparation of CTM medium, pH 8.5, which was used as the basal medium for growth of *C. thermophilum*. One liter of 50 × stock solution I contained 3.75 g magnesium sulfate (MgSO_4_ ⋅ 7H_2_O), 1.8 g calcium chloride (CaCl_2_ ⋅ 2H_2_O), 0.45 g sodium citrate, 0.5 ml trisodium-EDTA (stock: 89.6 g L^-1^, pH 8.0,), and 50 ml of the trace elements solution. Solution II contained 15.3 g potassium hydrogen phosphate per liter. Solution III contained 12 g of ferric ammonium citrate per liter. Solution IV contained 168.1 g of 2-oxoglutarate (sodium salt) per liter. Solutions I to IV were autoclaved prior to use. One liter of the trace elements stock solution contained: 2.86 g boric acid (H_3_BO_3_), 1.81 g manganese chloride (MnCl_2_ ⋅ 4H_2_O), 0.222 g zinc sulfate (ZnSO_4_ ⋅ 7H_2_O), 0.39 g sodium molybdate (Na_2_MoO_4_ ⋅ 2H_2_O), 0.079 g cupric sulfate (CuSO_4_ ⋅ 5H_2_O), and 0.0494 g cobaltous nitrate hexahydrate (Co(NO_3_)_2_ ⋅ 6H_2_O). A stock solution of vitamin B_12_ was prepared by adding 100 mg of cobalamin to 100 ml of double-distilled H_2_O, and the resulting solution was titrated with 1 M hydrogen chloride until the cobalamin dissolved (final pH 2.7). A 100-ml stock solution of 10 mM potassium phosphate buffer containing a mixture of 13 vitamins was prepared with 10 mg each of riboflavin and biotin and 100 mg each of thiamine hydrochloride, ascorbic acid, d-calcium-panthothenate, folic acid, nicotinamide, nicotinic acid, 4-aminobenzoic acid, pyridoxine hydrochloride, lipoic acid, nicotinamide adenine dinucleotide (NAD^+^), and thiamine pyrophosphate. The solution was titrated with 1 M sodium hydroxide until all compounds were dissolved (final pH 9.5). The two vitamin solutions were filter-sterilized through a 0.22-μm cellulose acetate filter and were stored in the dark at 4^∘^C until required.

One liter of growth medium was produced by mixing 20 ml of solution I, 3 ml of solution II, 2 ml of solution III, and 2.5 ml of solution IV, together with 2.4 g of HEPES and double-distilled H_2_O to a final volume of ∼970 ml. The medium was adjusted to pH 8.5 with 2 M potassium hydroxide and autoclaved at 121^∘^C for 40 min. The medium bottle was sealed immediately after autoclaving and cooled to ∼60^∘^C. The medium was finalized after cooling by adding 30 ml of a freshly prepared and filter-sterilized solution containing 0.125 g sodium thioglycolate, 0.625 g sodium bicarbonate, 1 ml of a Bacto^TM^ Peptone (BD Biosciences, Sparks, MD, USA) solution (stock: 100 mg ml^-1^), and 500 μl each of the vitamin B_12_ and 13-vitamin mixture solution was added. To produce a completely defined growth medium, Bacto^TM^ Peptone was replaced by a mixture of all 20 common l-AAs at a concentration of 5 mg L^-1^ each. Solidified medium additionally contained 1% (w/v) Bacto^TM^ Agar (BD Biosciences, Sparks, MD, USA) that had been washed three times with double-distilled H_2_O. Agar plates were filled with ∼50 ml of microoxic medium. Plates were incubated in the light in translucent sealed plastic jars (Becton Dickinson, Franklin Lakes, NJ, USA) that had been flushed with a 10% hydrogen/10% carbon dioxide/80% nitrogen (v/v/v) gas mixture to produce reduced oxygen conditions. Vessels used for experiments requiring liquid media were typically filled to ∼75% of the volume and were not shaken or mixed during growth experiments except for sampling. If not stated otherwise, the incubation temperature was 52.5^∘^C, the pH was 8.5, and the irradiance was 20–50 μmol photons m^-2^ s^-1^ provided by a tungsten bulb.

### High Performance Liquid Chromatography

Changes in concentrations of major nutrients (carbon, organic nitrogen and sulfur substrates) over time were followed by HPLC (UFLC module system, Shimadzu Scientific Instruments, Columbia, MD, USA). Carbon and organic sulfur substrates were analyzed using a SUPELCOGEL^TM^ C-610H column 59320-U (30 cm × 7.8 cm ID) and a SUPELGUARD C610H 5319 (5 cm × 4.6 mm ID) guard column (Supelco, Bellefonte, PA, USA). An isocratic elution protocol with 4 mM sulfuric acid as solvent and a total run time of 60 min and a flow rate of 0.5 ml min^-1^ were employed. The column oven had a temperature of 30^∘^C. Prior to injection of 20-μl aliquots of medium into the column, cells and debris were removed by centrifugation for 2 min at ∼12,800 × *g*. An aliquot (1.0 ml) of the supernatant was then additionally filtered through a 0.22-μm polytetrafluoroethylene filter prior to injection. Carbon compounds of interest were detected with a refractive index detector at 210 nm or with a UV/VIS detector. The identification of each analyzed substrate was confirmed using standard solutions of the corresponding substrates tested, and which were treated in the same way as the medium before analysis.

Changes in concentrations of AAs were also analyzed using the Shimadzu HPLC system. These analyses were performed with a Kinetex 5-μm C18 100Å column (15 cm × 4.6 mm ID) protected by a SecurityGuard ULTRA cartridge UHPLC C18 for 4.6-mm ID columns (Phenomenex, Torrance, CA, USA). AAs were derivatized with phenylisothiocyanate (PITC, Edman’s reagent) prior to detection at 254 nm. The derivatization procedure was based on the Thermo Scientific (Waltham, MA, USA) Example protocol for AA standard H, with some minor modifications. An aliquot (1.0 ml) of cell culture was centrifuged to remove cells and debris, and the resulting supernatant was evaporated at 55^∘^C under a stream of nitrogen gas. The dried sample was dissolved in 100 μl of coupling solution containing 5 μl of PITC, and the solution was incubated for 10 min at room temperature in the dark. The sample was again evaporated to dryness, and the residue was dissolved in 500 μl of solvent A and evaporated again. Finally, the dried sample was dissolved in 250 μl of solvent A and filtered through a 0.22-μm polytetrafluoroethylene filter prior to injection and analysis of a 20-μl aliquot of the solution. The HPLC analysis method consisted of a 2-solvent gradient developed over a 40-min period with a flow rate of 0.5 ml min^-1^. The initial condition was 100% solvent A, which decreased over 10 min to 82.5%, from 10–22 min to 80%, from 22–34.5 min to 30%, and from 35–40 min to 0%. Solvent A was 0.14 M sodium acetate, pH 6.2 containing 0.5 mM triethanolamine. Solvent B was a 40:60 (v/v) mixture of HPLC-grade water and acetonitrile. AAs were identified using AA standard mixture H (Thermo Scientific, Waltham, MA, USA). l-asparagine, l-glutamine, and l-tryptophan are not included in this standard mixture, and their elution times were established by analyzing each compound individually.

Pigment analyses of *C. thermophilum* strains were analyzed by a previously described method. Pigments were extracted from cells in acetone/methanol (7:2, v/v) and analyzed as described (Garcia Costas et al., 2012b).

### Bacterial Growth

Growth experiments were performed with liquid and/or solidified media. The cell inoculum was equivalent to 2% v/v of the fresh medium. Depending on the particular compound being tested, nutritional tests were made in duplicate or triplicate. Tests for essentiality of vitamins and AAs included up to four serial transfers with medium lacking the compound(s) being tested. The effects of different substrates on growth were tested for both the presence and the absence of the corresponding substrate.

Growth of *C. thermophilum* in liquid cultures was monitored by the Q_y_ band absorption of the BChl *c* at 667 nm in a Genesys^TM^ 10S UV/Vis scanning spectrophotometer (Thermo Scientific Rochester, NY, USA). An aliquot of cell culture (1.0 ml) was centrifuged at ∼15,000 × *g* for 4 min. The supernatant was removed and the cell pellet was resuspended in HPLC-grade methanol (1.0 ml) to extract the BChls. After 5-min incubation in the dark, the suspension was centrifuged for 2 min at ∼12,800 ×*g* and immediately analyzed.

### Oxygen Relationship/Requirement

The growth response of *C. thermophilum* to oxygen was tested with agar slants and plates at different oxygen concentrations [20% (atmospheric), 10%, 5%, and ∼0%, v/v] in the headspace of the medium, which was flushed for ∼1 min with the corresponding mixture of sterile nitrogen and oxygen. This was repeated every second day to ensure stable maintenance of the oxygen concentration. To produce an oxygen gradient in agar deeps, culture tubes were filled with CTM medium containing 1% (w/v) agar and were sealed with a headspace of air.

### Temperature and pH Range

To determine the optimal growth temperature and the temperature range over which growth could occur, cells were grown in CTM-Medium at pH 8.5 over the temperature range of ∼37–70^∘^C (±1^∘^C). To determine the optimal pH and the pH range over which growth could occur, cells were grown in CTM-medium from pH range 4–11 at 52.5^∘^C. The pH values of the medium were adjusted prior to autoclaving the medium and were measured again at the end of the experiment to ensure that the pH had not changed during the experiment.

### 16S rRNA Analyses

Genomic DNA was extracted according to the JGI-Standard protocol for DNA extraction of Gram-negative bacteria. Amplification was performed with a *C. thermophilum* specific forward primer (Cab f) and a universal reverse primer (1390r) which produced a ∼1300 bp 16S rRNA fragment using a standard PCR protocol as written elsewhere (Garcia Costas, 2010). Purified PCR products were sequenced with the Sanger DNA sequencing method using the same primers. Sequences were assembled, manually refined, and curated in SeqMan Pro Version 11 which is included in the Lasergene software package (DNASTAR, Madison, WI, USA). The 16S rRNA sequences were aligned with ClustalW prior determining the sequence similarities with DNAdist. Both are implemented in the BioEdit software program (Hall, 1999) and were used with default settings. The 16S rRNA sequences were deposited in GenBank under the accession numbers: KP300942- KP300947.

## Results and Discussion

### Historical Context for Growth and Isolation of *C. thermophilum*

Prior to the cultivation studies reported here that led to an axenic culture, *C. thermophilum* had been studied in several ways to gain information about this unusual bacterium. For example, the complete genome had been determined, and the photosynthetic apparatus including chlorosomes, BChl *a*-binding FMO protein, and type-1 photochemical reaction centers, as well as pigments, lipids and hopanoids, of *C. thermophilum* had been characterized in considerable detail (Bryant et al., 2007; Tsukatani et al., 2010, 2012; Wen et al., 2011; Garcia Costas et al., 2012a,b). These studies classified *C. thermophilum* as an anoxygenic, chlorophototrophic Acidobacterium, and the data strongly indicated that this organism relies on organic carbon source(s) (i.e., branched chain AAs), reduced sulfur source(s) and oxygen for BChl, carotenoid, and tyrosine biosynthesis. In spite of this considerable knowledge, the enrichment cultures of *C. thermophilum,* although relatively stable, only produced low and variable cell yields. The two heterotrophic contaminants severely limited further progress on biochemical, metabolic, and physiological studies.

Discoveries and descriptions of new bacteria often occur in a simple and familiar manner. A newly acquired sample is inoculated into a previously known growth medium, and those organisms that grow are screened for representatives of new species. Potentially interesting representatives are rendered axenic and are initially characterized using the same growth medium. The situation was completely different for *C. thermophilum*. *C. thermophilum* could be grown under non-optimal conditions, but almost nothing was known about the specific nutrients and growth conditions that would be required to grow this bacterium axenically. Although bacteria have considerable metabolic versatility, they typically require only a limited number of macronutrients, to provide major elements (carbon, hydrogen, nitrogen, oxygen, phosphorus, and sulfur) as well as trace minerals, vitamins, or other growth factors. The challenge in this case was to establish how to provide the right substrates at the right concentrations under the right physico-chemical conditions to allow the growth *C. thermophilum*. As a starting point in obtaining an axenic culture of *C. thermophilum,* we established that phosphate, trace elements and vitamins (other than vitamin B_12_; see below) did not negatively affect the growth of *C. thermophilum*, and thus the concentrations of these nutrients were not varied during the testing that ultimately led to the axenic culture. With the exception of vitamins (see below), changes in the concentrations of these substrates had little or no effects on the growth of axenic culture of *C. thermophilum.* Thus, the main focus was to identify substrates that could serve as sulfur, carbon, and nitrogen sources, respectively. Secondarily, the role of oxygen and the optimal pH and temperature conditions had to be determined.

Because *C. thermophilum* is the first anoxygenic chlorophototrophic member of a poorly characterized and highly diverse phylum, *Acidobacteria*, there were no comparable organisms that could provide guidance for its purification and cultivation. Genomic data were screened for the presence and absence of key metabolic pathways, intermediates, and transporters/permeases using the Kyoto Encyclopedia of Genes and Genomes (KEGG; http://www.genome.jp/kegg/). Promising candidate substrates (as described below) were tested in cultivation experiments, and the outcomes of these cultivation experiments were refined and used for an amended characterization of *C. thermophilum*, which ultimately led to the establishment of a defined medium.

### Sulfur Sources

**FIGURE 1:**
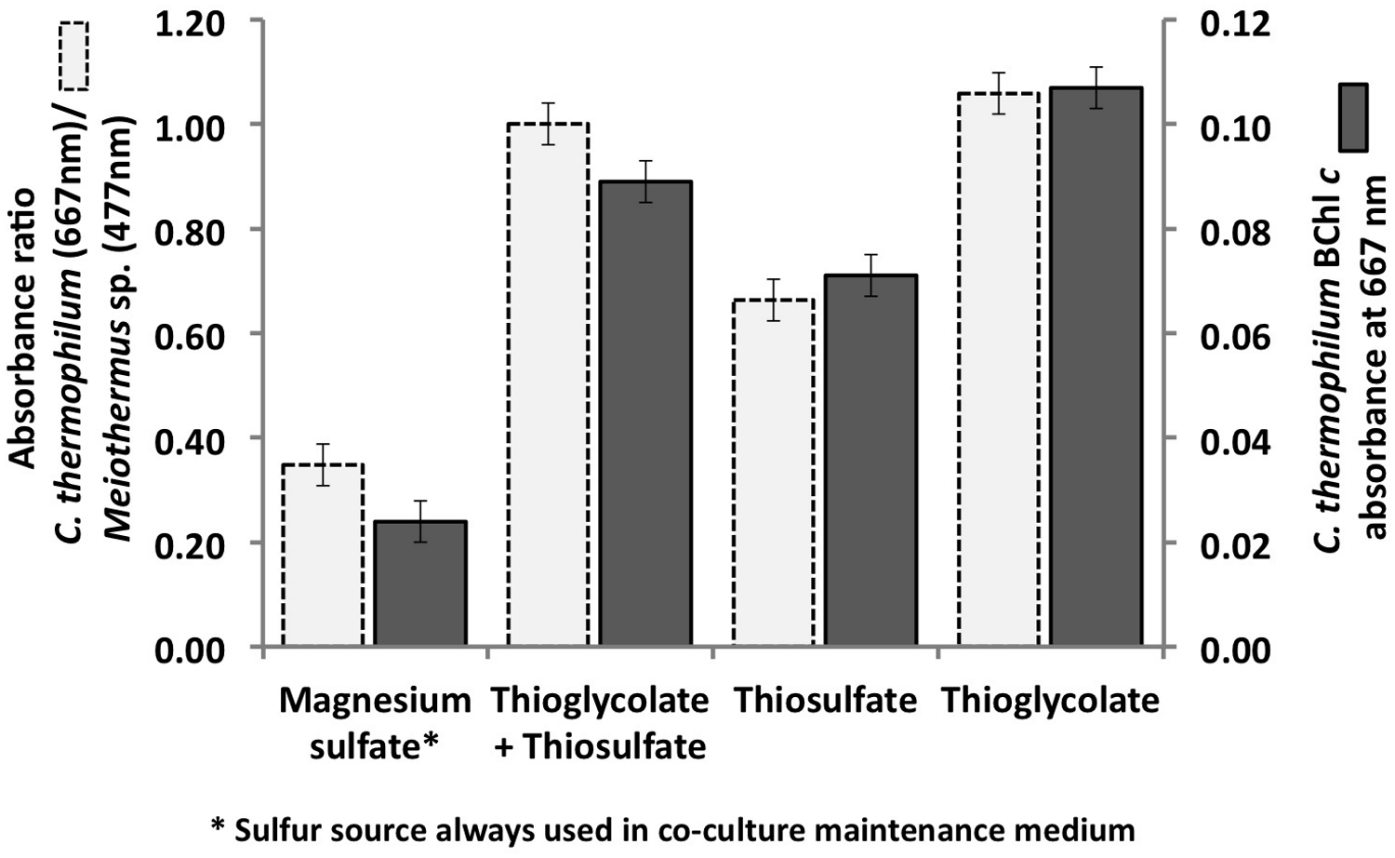
**Initial growth tests performed with the co-culture of *C. thermophilum* strain B^**T**^, *Meiothermus* sp. and *Anoxybacillus* sp. in order to identify suitable reduced sulfur sources for *C. thermophilum* strain B^**T**^**. Magnesium sulfate served as sulfur source in the maintenance medium for the co-culture (left bars; see text for additional details). Sulfate was replaced by thioglycolate, thiosulfate, or both in the medium for the axenic strain. Measurements were taken on day 8 after inoculation.

**FIGURE 2:**
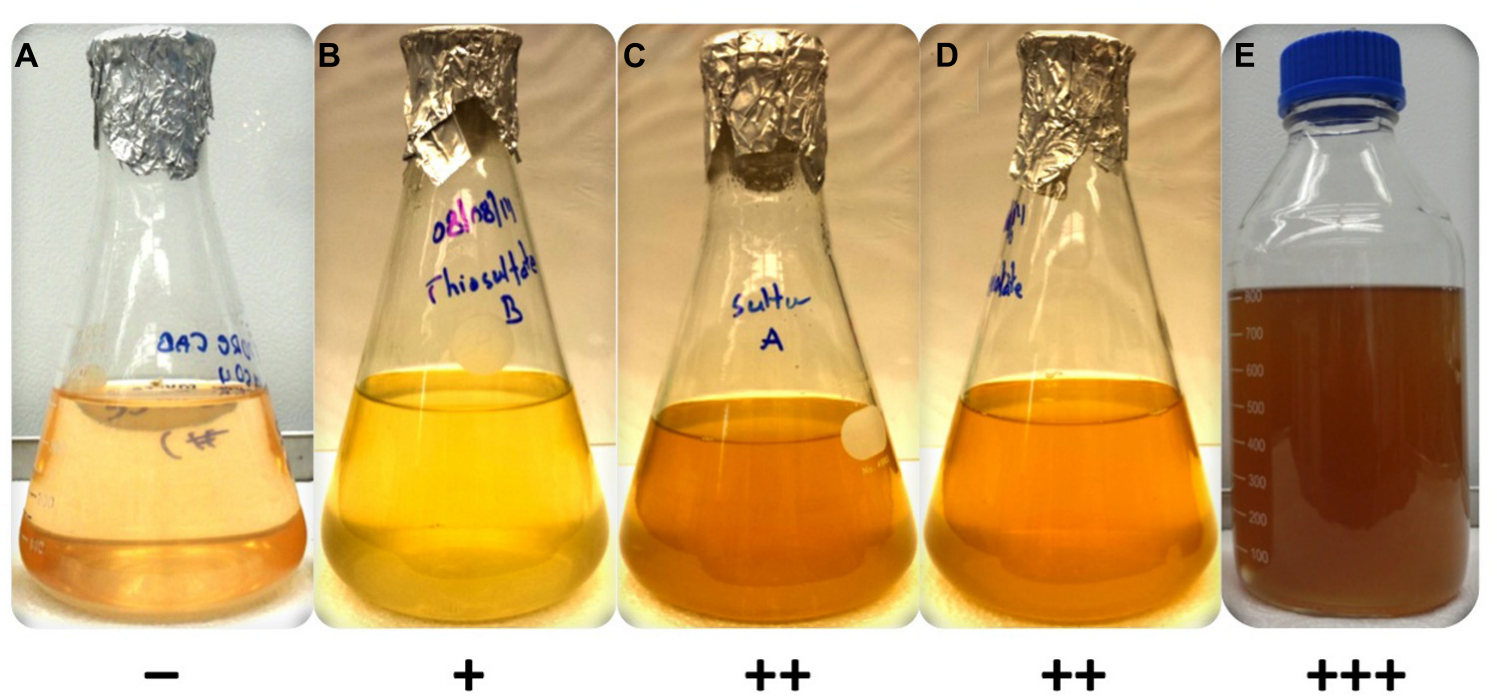
**Growth of axenic culture of *C. thermophilum* strain B^T^ on different sulfur sources in CTM-medium**. Growth occurred only in the presence of a reduced sulfur source **(B–E)**. *C. thermophilum* strain B^T^ did not grow with magnesium sulfate **(A)** as it relies on reduced sulfur compounds. The reduced sulfur substrates tested were **(B)** sodium thiosulfate, **(C)** sulfur **(D)** sodium thioglycolate and **(E)** l-methionine/l-cysteine. The symbols at the bottom reflect a qualitative assessment of growth of *C. thermophilum* as assessed by BChl *c* synthesis.

As predicted from the genomic sequence data (Garcia Costas et al., 2012a), *C. thermophilum* was unable to use sulfate as a sulfur source because it does not have the genes for enzymes of assimilatory sulfate reduction. *C. thermophilum* instead uses reduced sulfur sources. Adding reduced sulfur sources to co-cultures of *C. thermophilum* and the two heterotrophs considerably improved the growth of *C. thermophilum* (**Figure 1**). Thioglycolate produced the greatest growth enhancement, and therefore it was used as the preferred sulfur source in a revised medium. Subsequent growth tests with axenic cultures of *C. thermophilum* reconfirmed the need for reduced sulfur sources for growth. Doubling the thioglycolate concentration clearly retarded or sometimes even inhibited the growth of *C. theromphilum*; this may have occurred because of depletion of dissolved oxygen by the reaction of the thiologlycolate with oxygen. Axenic cultures of *C. thermophilum* also grow well with l-methionine and l-cysteine/cystine as reduced sulfur sources (**Figure 2**). Thiosulfate and elemental sulfur can also serve as the sulfur source, but lower cell yields were obtained with these compounds. The use of sulfide by *C. thermophilum* is enigmatic. Cultures containing sodium sulfide did not show sustained growth, but microscopic analyses showed that sulfur globules were produced. Similar to green sulfur bacteria, these globules remained associated with the outer surfaces of cells, and suggested that sulfide oxidation occurred (data not shown). The genome lacks any known enzymes for the oxidation of sulfide, so how sulfide oxidation occurs is not clear.

### Carbon Sources

**FIGURE 3:**
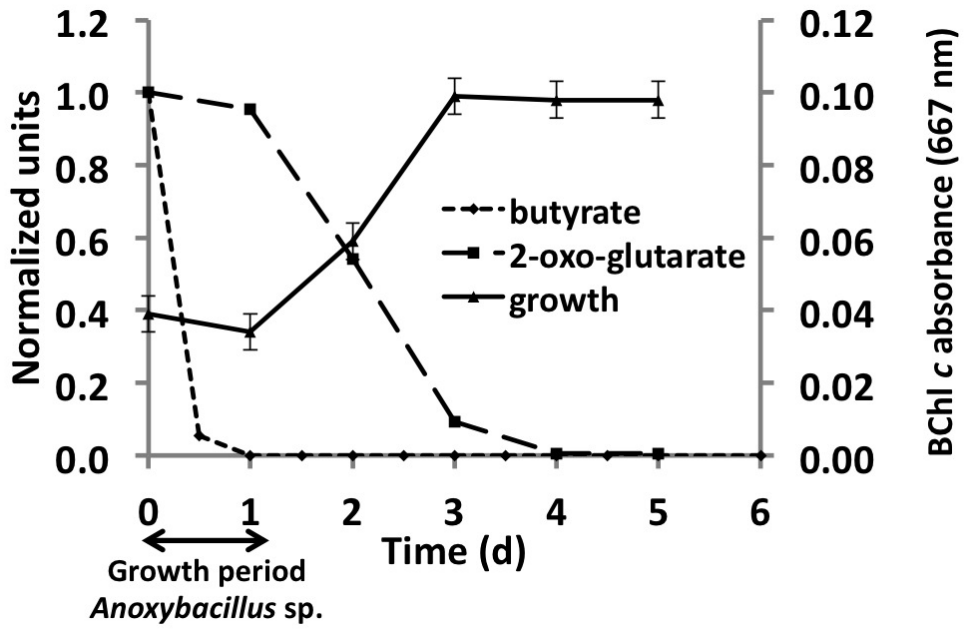
**Correlation between growth of *C. thermophilum* strain B^**T**^ and the consumption of 2-oxoglutarate and butyrate in a co-culture with *Anoxybacillus* sp**. At the end of the initial growth period (∼24 h) when butyrate was completely consumed, only spores of *Anoxybacillus* sp. were observed. After *Anoxybacillus* sp. had sporulated, consumption of 2-oxoglutarate commenced and concomitantly, the BChl *c* concentration in the medium, indicative of the growth of *C. thermophilum,* increased until the 2-oxoglutarate was consumed.

Analyses of the *C. thermophilum* genome clearly indicated that this bacterium should be a photoheterotroph, because key enzymes belonging to all known CO_2_ fixation pathways were missing. Therefore, we focused on the search for suitable organic carbon sources, and we began by testing each of the compounds used in the co-culture medium individually. Acetate, butyrate, citrate, glycolate, pyruvate, lactate, and succinate were all added to the co-culture medium, because they are compounds that have been detected, or are expected to be present, in the microbial mats from which the *C. thermophilum* was enriched (Anderson et al., 1987; Bateson and Ward, 1988; also see Kim et al., 2015). The best growth yields of *C. thermophilum* were achieved with butyrate (10 mM), followed by acetate and succinate as single carbon sources in these initial tests. Although this had seemed to be a logical and reasonable place to start cultivation studies—and indeed a stable co-culture was maintained for several years on this mixture of compounds—*C. thermophilum* actually does not use any of these seven carbon sources. **Figure 3** shows data for butyrate, and these results were eventually verified by retesting each of these compounds with the axenic culture. When the consumption of these potential carbon sources was monitored by HPLC, their disappearance was actually correlated with growth of *Anoxybacillus* sp. and *Meiothermus* sp. (data not shown) but not with *C. thermophilum* (see **Figure 3**). Because the kinetics of the appearance of BChl *c* in the cultures did not match the kinetics of disappearance of any of the seven initial carbon substrates (i.e., acetate, butyrate, citrate, glycolate, pyruvate, lactate, and succinate), we tested 2-oxoglutarate as an alternative growth substrate. When 2-oxoglutarate was added together with one or more of the other carbon substrates, consumption of the 2-oxoglutarate began about 24 h after fresh growth medium was inoculated with the mixture of cells (**Figure 3**), and the accumulation of BChl *c* (i.e., *C. thermophilum* cells) ceased when the 2-oxoglutarate was depleted from the growth medium. Because it improved the growth of *C. thermophilum* in the co-cultures, 2-oxoglutarate was an important substrate on the way to obtaining an axenic culture of *C. thermophilum*. However, it is not essential for growth of *C. thermophilum*, because growth still occurred without added 2-oxoglutarate in subsequent experiments with axenic cultures (data not shown).

**FIGURE 4:**
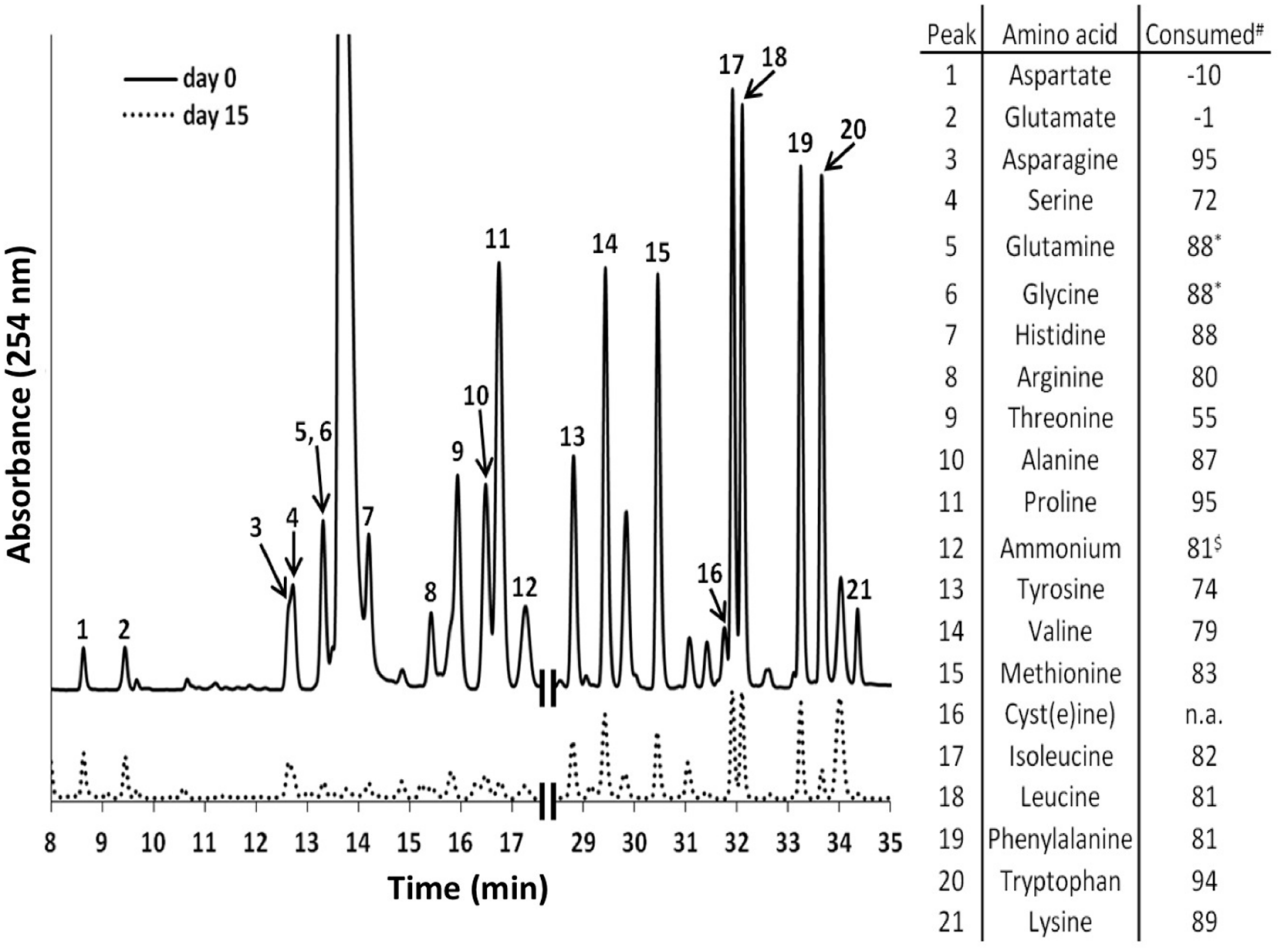
**High performance liquid chromatography elution profiles of amino acids (AA) in the growth medium of *C. thermophilum* strain B^**T**^**. The solid line shows the HPLC elution profile of the reference medium with all 20 AAs (5 mg L^-1^ of each) just prior to inoculation with *C. thermophilum* cells. The dashed line shows an elution profile for the spent medium after 15 days of growth when 19 AAs (all except l-cysteine) was added. The numbered peaks are identified in the table at the right, which shows the amount of each AA that had been consumed after 15 days. The table at the right shows the consumption or production (negative numbers for aspartate and glutamate) of each AA, which are based upon the peak heights differences between day 0 and day 15. Glutamine and glycine were not separated in our standard elution protocol and thus were calculated together. n.a., not added. $, free ammonium. Note that all AAs added were consumed except aspartate and glutamate.

Additional growth experiments showed that bicarbonate and the AAs l-isoleucine, l-leucine, l-lysine, and l-valine are essential for growth of *C. thermophilum* (also see the section on nitrogen sources below). *C. thermophilum* did not grow when bicarbonate was omitted from the growth medium. We hypothesize that bicarbonate may be used in anaplerotic reactions to replenish the pool of certain required organic molecules (Tang et al., 2011). However, photoautotrophic growth with bicarbonate as the sole carbon source was never observed. HPLC analyses of culture medium showed that *C. thermophilum* can take up and utilize all AAs except aspartate and glutamate. Their disappearance from the medium could be correlated with the growth of *C. thermophilum* in experiments with the axenic culture (see **Figure 4**). However, it still needs to be determined whether the AAs are used primarily as carbon sources, nitrogen sources, or both.

**FIGURE 5:**
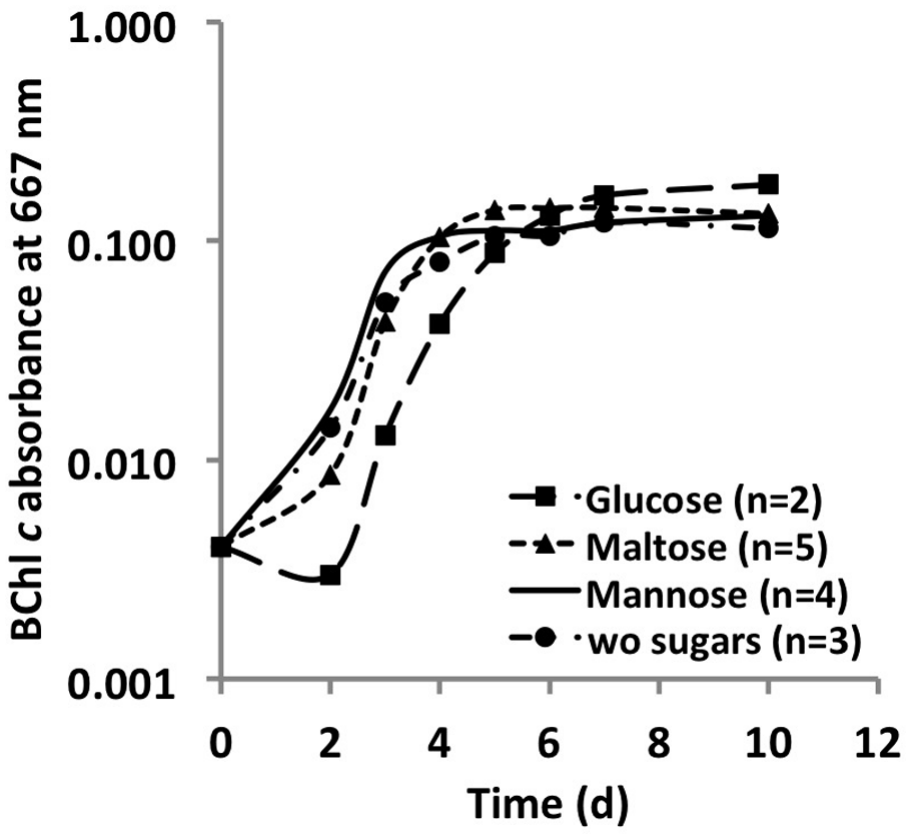
**Growth curves of *C. thermophilum* strain B^T^ from sugar utilization tests in liquid culture**. Growth is plotted as a function of BChl *c* absorbance at 667 nm over time.

Monosaccharides, disaccharides and polysaccharides, including mannose, glucose, fructose, rhamnose, maltose, glycogen, starch, cellulose, and chitin, were tested as additional carbon sources. Only mannose, glucose and maltose showed any effect on the growth of *C. thermophilum*. Compared to cells grown in their absence, when cells were grown in a medium containing one of these three sugars, the cells showed an obvious increase in size (data not shown). However, distinct growth rate differences in sugar-supplemented medium compared to sugar-free medium were not observed based on BChl measurements (**Figure 5**). Because the sugars were only depleted by 20–35% from the growth medium, their effects on the growth of *C. thermophilum* are still unclear. Other organic carbon sources, including pyruvate, fumarate, malate, oxaloacetate, formate, ethanol, and methanol, did not produce any clear effects on the growth of *C. thermophilum*. The BChl contents of cultures containing these compounds did not differ significantly from those of control cultures. We thus conclude that *C. thermophilum* is a nutritional specialist that is restricted to only a few carbon sources, principally AAs, and that it requires four AAs specifically as noted above.

### Nitrogen Sources

**FIGURE 6:**
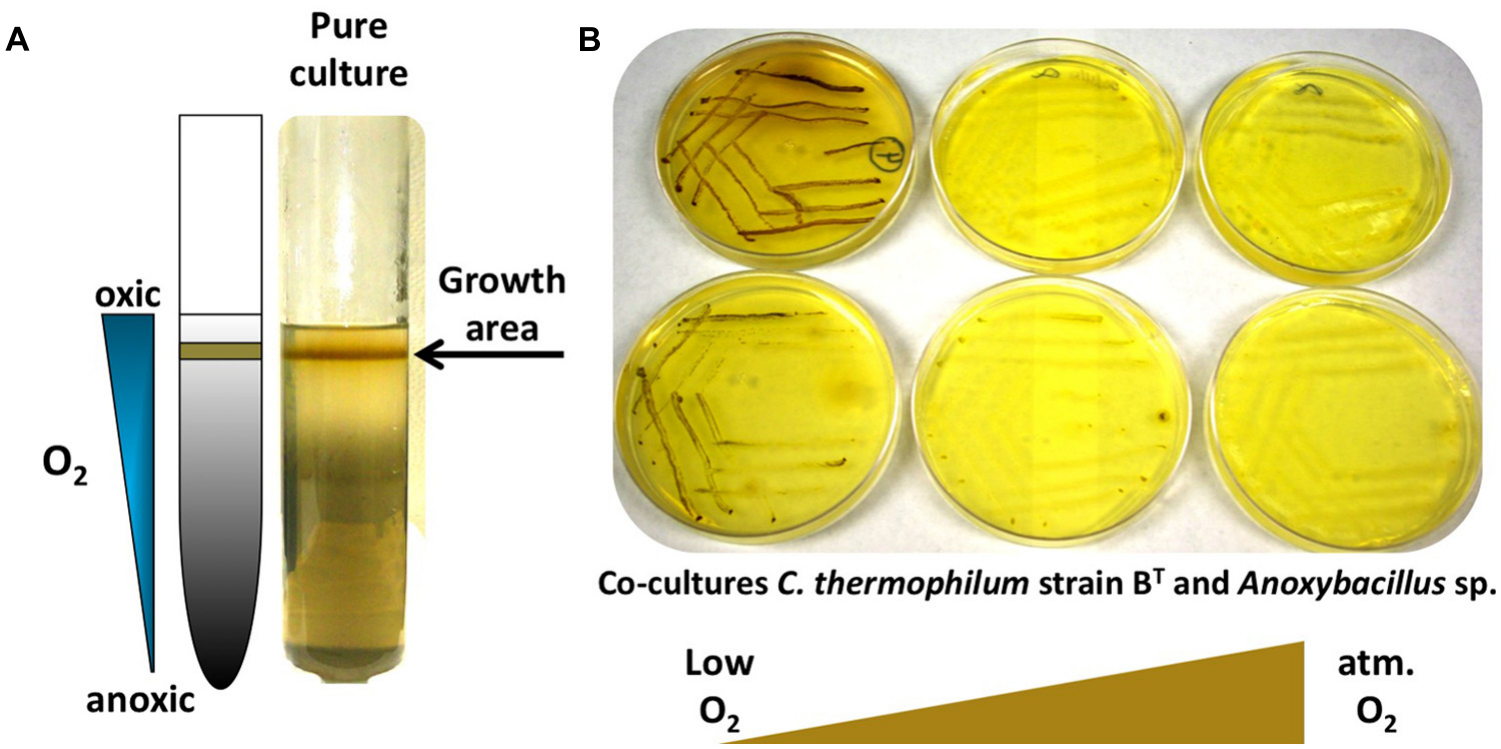
**Growth of *C. thermophilum* in the presence of oxygen**. **(A)** *C. thermophilum* strain B^T^ cultivated in an agar shake with a naturally established oxygen gradient demonstrating preferential growth under microoxic conditions. **(B)** Cultivation of co-culture of *C. thermophilum* strain B^T^ and *Anoxybacillus* sp. streaked on agar plates at different oxygen concentration resembling low to atmospheric oxygen tension. Establishing a lowered oxygen environment was the crucial step in the axenic isolation of *C. thermophilum* strain B^T^.

The *C. thermophilum* genome does not contain genes for nitrogenase or assmililatory nitrate or nitrite reduction, and thus, not surprisingly, no growth occurred with dinitrogen or nitrate as sole N-source. Unexpectedly, *C. thermophilum* was also unable to grow with ammonium as the sole N-source. This was surprising because the genome encodes a gene predicted to encode an ammonium transporter (*amtB*; Cabther_A0161). In fact, *C. thermophilu*m cells lysed in the presence of 1 mM ammonium. Because genome analyses also predicted putative transporters for branched chain AAs, we started to use yeast extract (100 mg L^-1^) as the nitrogen source in the growth medium. Together with the identification of a suitable S-source (thioglycolate) and carbon source (bicarbonate and 2-oxoglutarate), this N-source enabled the growth of *C. thermophilum* on plates. *C. thermophilum* could easily be separated from the *Meiothermus* sp. by using the improved medium, but *C. thermophilum* still grew in tight association with *Anoxybacillus* sp. (**Figure 6B**), which could be eliminated after discovering the oxygen sensitivity of *C. thermophilum* (see discussion of Oxygen Relationships below).

**FIGURE 7:**
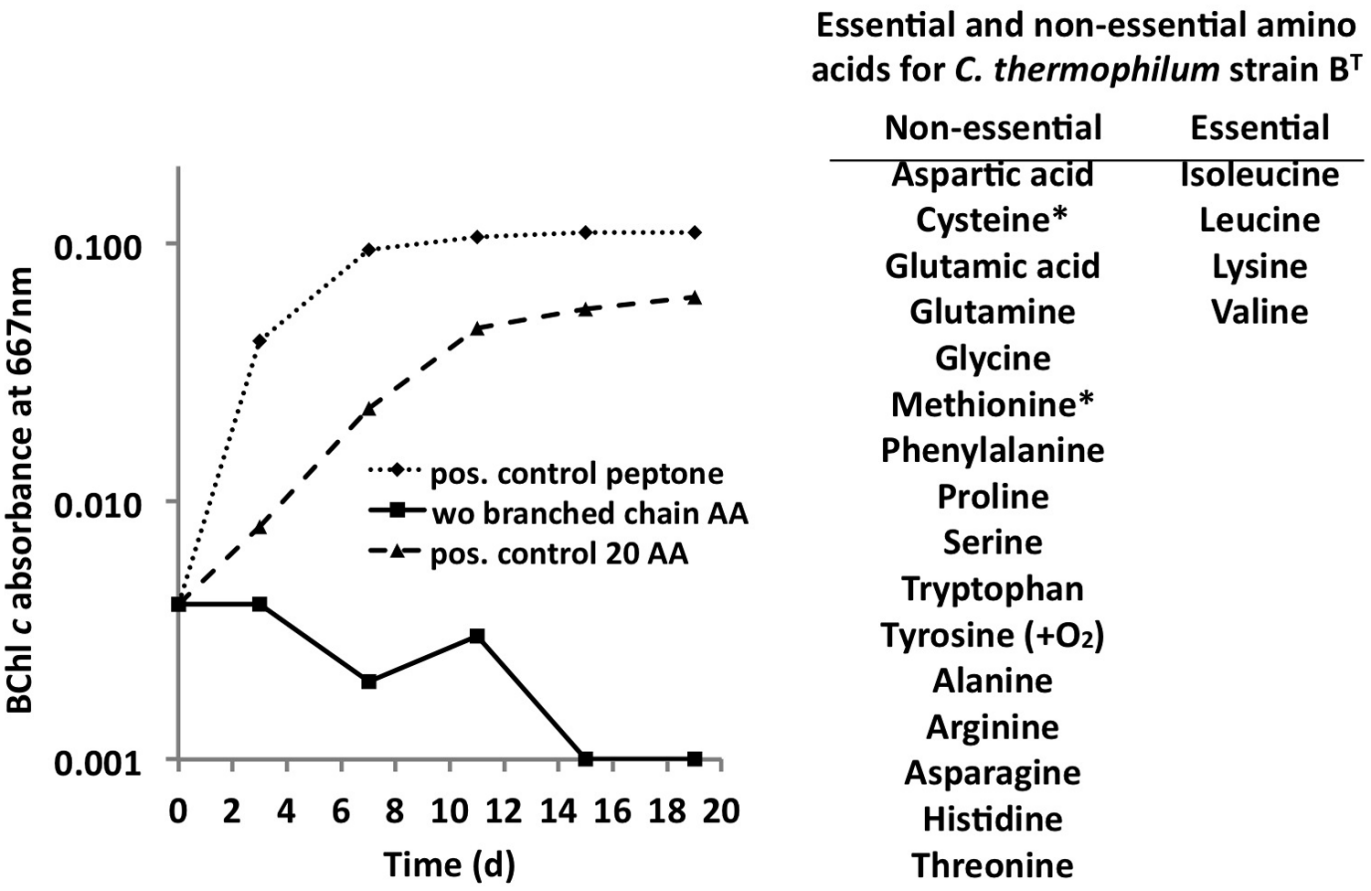
**Branched chain AAs are essential for growth of *C. thermophilum***. Growth of *C. thermophilum* strain B^T^ with peptone, 20 common AAs and common AA without the branched chain AAs l-isoleucine, l-leucine, and l-valine. Note that no growth occurred in the absence of branched chain AAs. The asterisks for cysteine and methionine indicate that one of these AAs is essential in the absence of a reduced sulfur source. Tyrosine is essential under very low oxygen concentrations.

**FIGURE 8:**
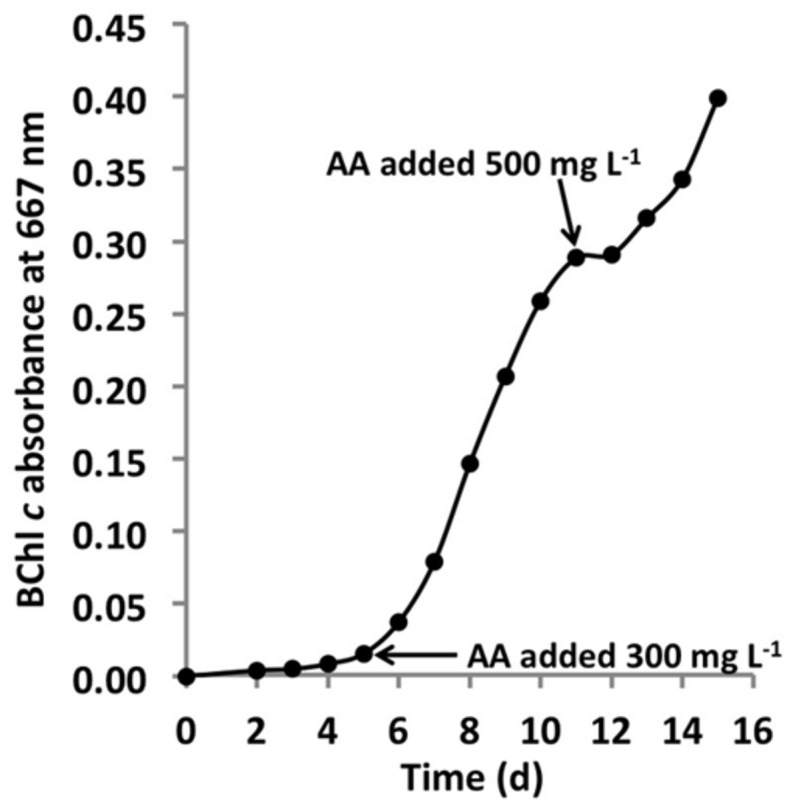
**Growth stimulation by addition of AAs**. Growth of *C. thermophilum* strain B^T^ in CTM-medium that was supplemented two times with a mixture of the 20 common AA, at concentrations of 300 mg L^-1^ and 500 mg L^-1^ as indicated by the arrows. Note, that each addition of AAs (arrows enhanced) growth. *C. thermophilum* strain B^T^ typically reaches stationary phase at BChl *c* absorbance values of 0.1–0.15 without supplemental feeding.

*C. thermophilum* grew well on plates and in liquid culture when yeast extract was replaced by 100 mg L^-1^ peptone, which confirmed that *C. thermophilum* requires AAs for growth. In order to produce a completely defined growth medium for *C. thermophilum,* and to determine which AAs are utilized by *C. thermophilum,* we conducted growth experiments with axenic cultures, in which we tested different combinations of AAs, e.g., alpha-keto acids of the branched chain AAs plus ammonium, branched chain AAs only, and AAs without branched chain AAs. We also tested the essentiality of all 20 AAs individually. Growth of *C. thermophilum* is strictly dependent upon the branched chain AAs, l-isoleucine, l-leucine, and l-valine (**Figure 7**), and interestingly, l-lysine is also essential. When these four AAs were omitted from any growth medium, minimal or no growth of *C. thermophilum* occurred after the first transfer. The essentiality of l-isoleucine, l-leucine, l-lysine, and l-valine is consistent with the genomic sequence data that had predicted that *C. thermophilum* would be unable to synthesize these four AAs (Garcia Costas et al., 2012a). On the other hand *C. thermophilum* still grew after the third transfer into medium that contained these four AAs as sole nitrogen source, confirming that these AAs are capable of providing all nitrogen required for growth. All other AAs can be synthesized by *C. thermophilum,* which could be demonstrated in experiments in which one or more of the other sixteen AAs were omitted. In addition, HPLC analyses of AA utilization revealed that *C. thermophilum* is able to metabolize at least 18 of the 20 common AAs (**Figure 4**). Over a period of 14 days all AAs were consumed to varying extents except aspartic acid and glutamate, which actually seemed to be produced and excreted rather than being consumed. AA uptake ranged from a minimal value of 55% (l-threonine) to ∼95% (l-proline). As observed for the S- and C-sources, the response to N-source of *C. thermophilum* showed an oligotrophic behavior that is common among Acidobacteria (Eichorst et al., 2007; Fierer et al., 2007). Growth was not improved by simply adding a higher concentration of AAs to the medium at the start of cultivation. However, cultures produced higher biomass when they were supplemented with AAs over the time course for growth (**Figure 8**) Although they are common, natural, nitrogen-rich substances, putrescine, betaine, and DNA were not utilized as N-sources by *C. thermophilum*.

### Oxygen Relationship

Establishing the growth relationship of *C. thermophilum* to oxygen was one of the biggest challenges, but studies with oxygen provided one of the key insights that led to an axenic culture. Growth tests clearly demonstrated that *C. thermophilum* preferred low oxygen concentrations for growth and maintenance in the laboratory. This correlates well with knowledge that *C. thermophilum* does not grow near the surface of the microbial mat community from which it is derived but that it grows near the bottom of the photic layer of these mats (Liu et al., 2012). No growth occurred under anoxic conditions (in a chamber with an atmosphere of H_2_, CO_2_, and N_2_ (10:10:80 vol/vol/vol)), or in fully oxygenated cultures that were vigorously shaken. *C. thermophilum* showed the typical growth pattern observed for microaerophiles in agar deeps (**Figure 6A**). Growth only occurred in the narrow interface between the oxic and anoxic regions of the agar deep. Interestingly, *C. thermophilum* survived long-term exposures to both fully oxic and anoxic conditions, but survival was distinctly longer in oxygenated medium. Because of the technical difficulty of providing alternating microoxic and anoxic conditions, we did not test if *C. thermophilum* could grow better under alternating oxygen concentrations, as occurs over each diel cycle in its natural habitat (Liu et al., 2011, 2012). In contrast to other bacteria that contain homodimeric type-1 reaction centers (green sulfur bacteria and heliobacteria; Bryant and Liu, 2013), which have reaction centers that are highly sensitive to oxygen, *C. thermophilum* requires oxygen for the biosynthesis of (B)Chls and carotenoids and also requires oxygen for the synthesis of tyrosine from phenylalanine (Garcia Costas et al., 2012a).

### Vitamins

**FIGURE 9:**
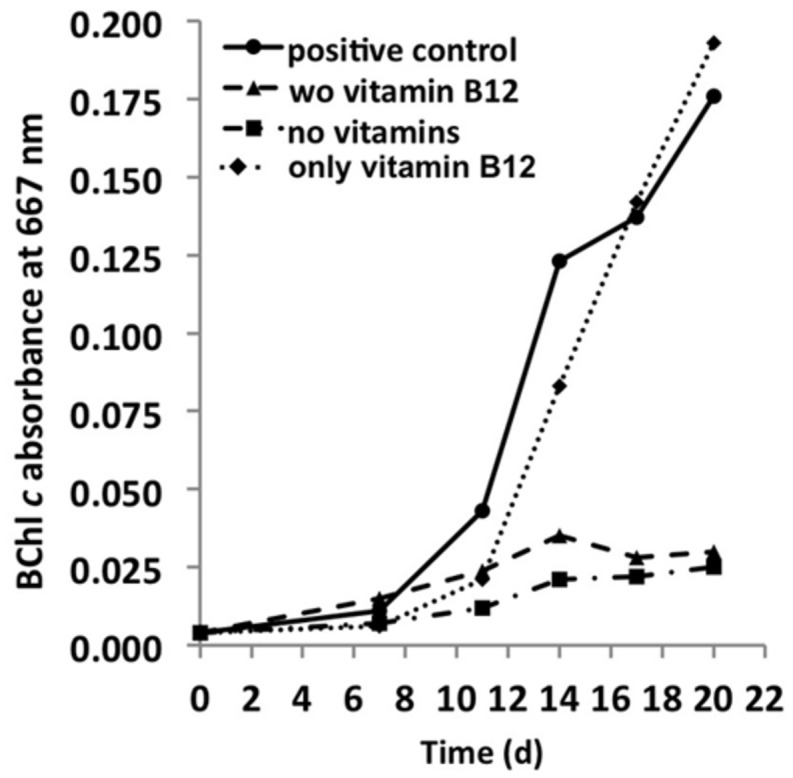
**Vitamin requirements of *C. thermophilum* strain B^**T**^**. The chart shows the growth with different combinations of vitamins after second transfer. Cultures containing Vitamin B_12_ show normal growth. Cultures in medium lacking vitamin B_12_ already show very weak growth after the second transfer. Thus, *C. thermophilum* only requires vitamin B_12_.

The genome predicts that *C. thermophilum* requires vitamin B_12_ for l-methionine synthesis (Garcia Costas et al., 2012a), and because most of the genes for vitamin B_12_ synthesis are missing in the genome, it was not surprising to establish that vitamin B_12_ was essential for maintaining the growth of *C. thermophilum*. Reduced growth in the absence of vitamin B_12_ was obvious after the second transfer of cells into medium free of vitamin B_12_ (**Figure 9**). Cells starved for vitamin B_12_ exhibited very weak fluorescence from BChls by epifluorescence microscopy (data not shown). This is consistent with the important role that S-adenosylmethionine plays in Chl biosynthesis in general and biosynthesis of BChl *c* methylation homologs specifically (Bryant et al., 2007; Garcia Costas et al., 2011). When the 13-vitamin mix was omitted from the growth medium no obvious effect on growth was noted after four serial transfers into medium free of vitamins other than vitamin B_12_. This observation confirms that vitamin B_12_ is the only vitamin required for growth of *C. thermophilum*.

### Light Requirements

*C. thermophilum* is a phototrophic bacterium that produces chlorosomes, and it showed the best growth under low continuous irradiance, ∼20–50 μmol photons m^-2^ s^-1^ (from a tungsten source). Light intensities above 50 μmol photons m^-2^ s^-1^ led to cell lysis. Very weak growth occurred in medium containing 2.5 mM 2-oxoglutarate and 2.5 mM mannose (together with AAs) in the dark, but otherwise, growth was dependent upon light in all experiments. Several explanations for the preference for lower irradiance can be given. Like green sulfur bacteria and green filamentous anoxygenic phototrophs like *Chloroflexus* sp., *C. thermophilum* produces chlorosomes (Bryant et al., 2012). Chlorosomes are the most efficient light harvesting organelles in nature (Frigaard and Bryant, 2006), and they have evolved to allow bacteria to obtain sufficient energy for phototrophic metabolism under very low irradiance conditions. Because *C. thermophilum* obviously does not generate biomass from CO_2_ fixation and does not fix nitrogen, both of which are energy intensive processes, the energy needed from light is not particularly high and the metabolism of organic molecules may also serve as an energy source via respiration. All genes required to produce an aerobic respiration chain are found in the genome (Garcia Costas et al., 2012a). A third reason may be that too many reactive oxygen species are produced at high irradiance conditions, and these could damage the phototrophic apparatus of *C. thermophilum*.

### Temperature and pH Range and Optima

**FIGURE 10:**
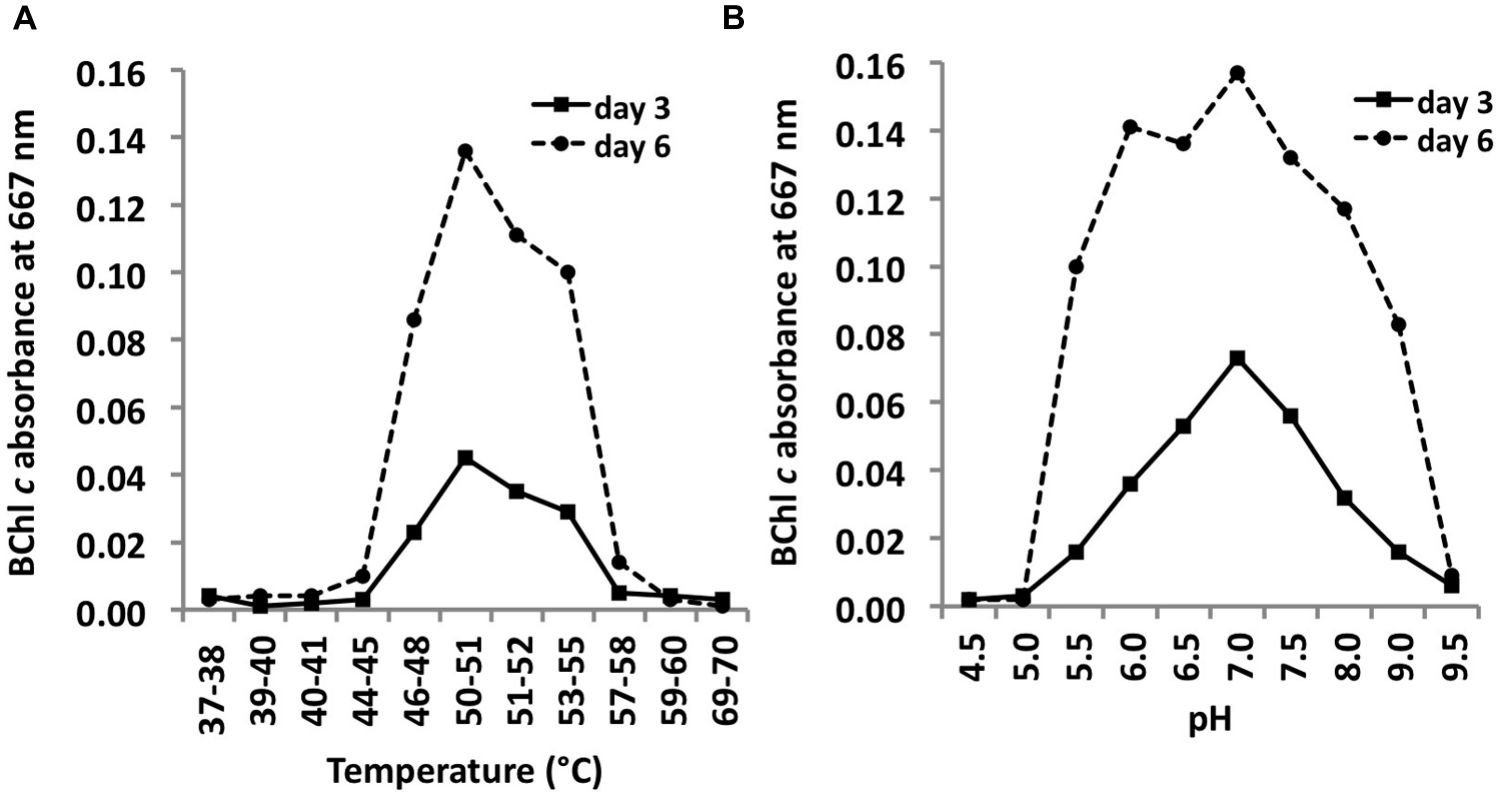
**Temperature and pH optima for growth**. Temperature **(A)** and pH ranges **(B)** for growth of *C. thermophilum* strain B^T^. Cells were grown in CTM-Medium, pH 8.5 for 6 days, and relative cell yields on day 3 and day 6 were determined by the amount of BChl *c* that was synthesized (see Materials and Methods). Optimal growth was observed at 51–52^∘^C; a broad pH optimum centered at about pH 7 was observed.

*C. thermophilum* strain B^T^ (Tank and Bryant, 2015) grew at temperatures between 44 and 58^∘^C with a T_opt_ of ∼51^∘^C (**Figure 10A**). This relatively narrow temperature range supports the findings from Miller et al. (2009) that different ecotypes of *C. thermophilum* are adapted to specific temperatures. Miller et al. (2009) found *C. thermophilum* growing at temperatures of 38–68^∘^C in White Creek and Garcia Costas (2010) found *C. thermophilum* in several mat samples collected at temperatures from 34 to 68^∘^C in Yellowstone National Park, WY, USA. *C. thermophilum* can be classified as a moderately thermophilic bacterium.

*C. thermophilum* grew between pH 5.5 and 9.5 and exhibited a broad optimum at circum-neutral pH (**Figure 10B**). *C. thermophilum* is well adapted to the pH value of its *in situ* habitat, which ranges from circum-neutral in the morning to around 9.5 in the late afternoon in the microbial mats at Mushroom and Octopus Spring (Jensen et al., 2011).

### Isolation of New *C. thermophilum* Strains

From previous studies conducted at different hot springs in Yellowstone National Park, it was known that different representatives of *C. thermophilum* with different temperature optima occur in these chlorophototrophic microbial mats (Miller et al., 2009; Garcia Costas, 2010; Ross et al., 2012). More distantly related sequences 16S rRNA sequences have been recovered from microbial communities associated with hot springs in Tibet and Thailand (Kanokratana et al., 2004; Yim et al., 2006; Lau et al., 2009). These facts encouraged us to test the suitability of our defined CTM-medium, together with the experimental growth conditions used for lab strain B^T^, to isolate new *C. thermophilum* strains directly from the environment.

**FIGURE 11:**
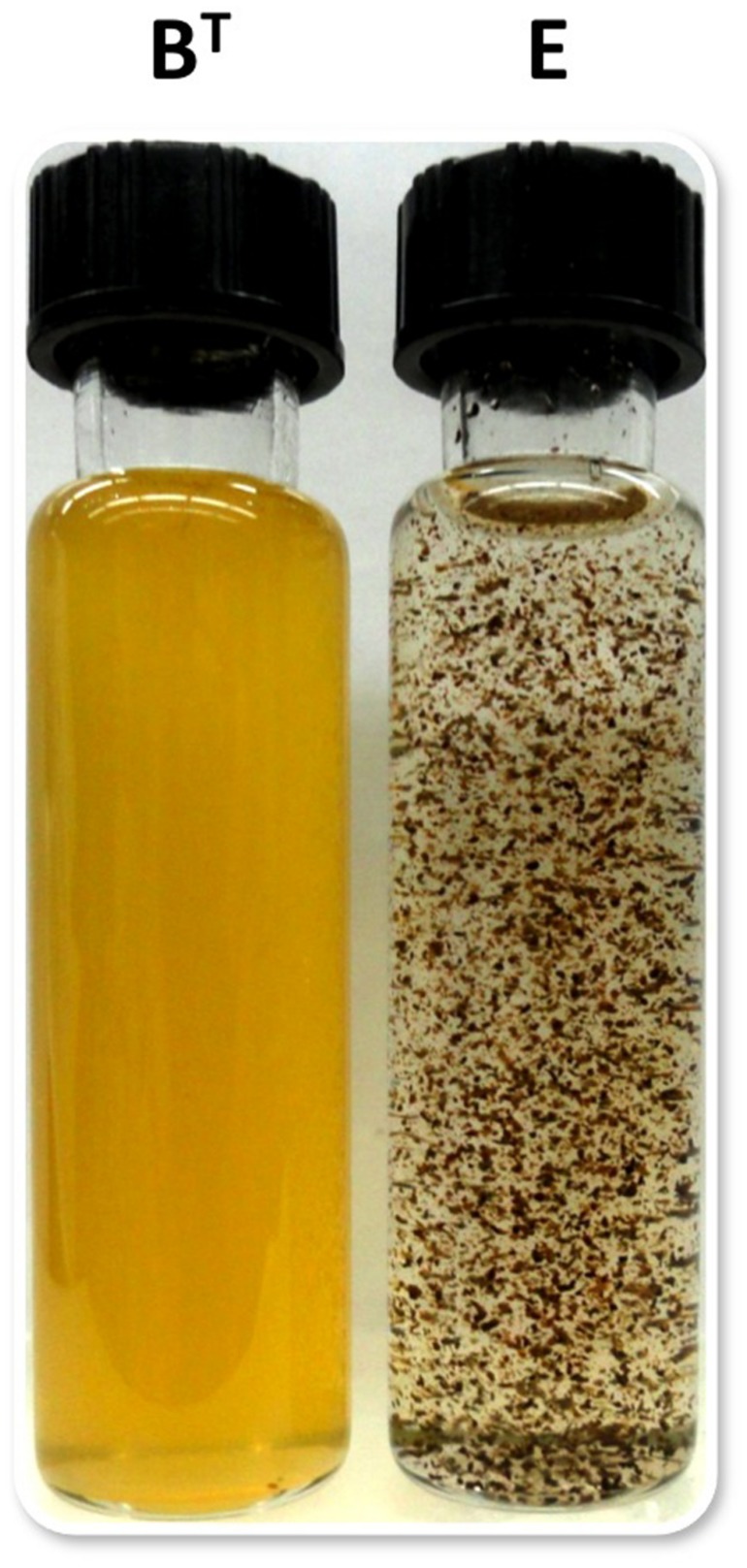
**Appearance of liquid cultures of *C. thermophilum* strain B^T^ and strain E**. Note the cell aggregates and clumpy growth of strain E compared to the homogeneous cell suspension of strain B^T^. Both cultures were shaken prior imaging. Cells of *C. thermophilum* do not float during growth but settle to the bottom of growth vessel.

**FIGURE 12:**
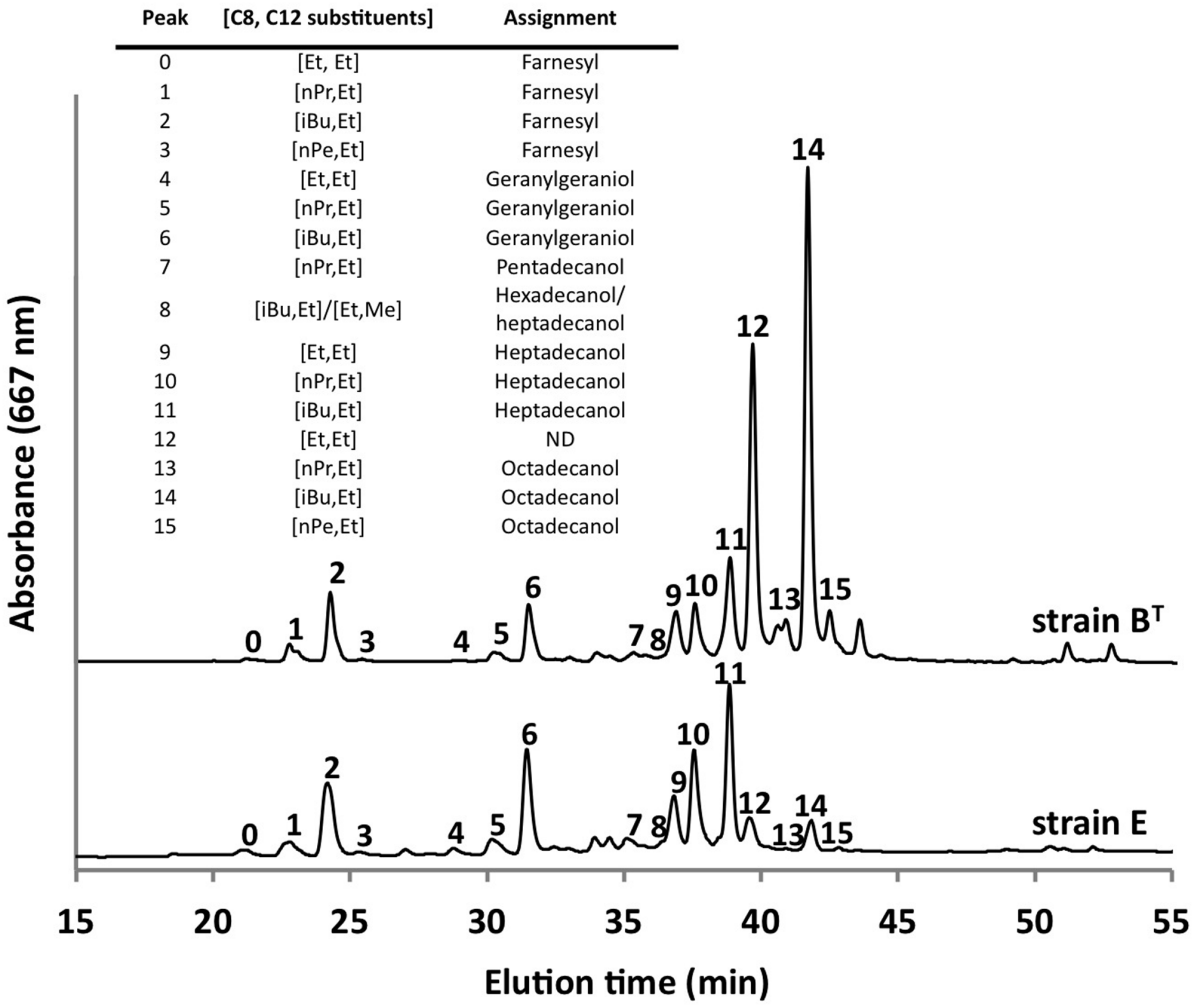
**Comparison of BChl *c* homologs in *C. thermophilum* strain B^T^ and strain E**. Both strain were cultivated under the same growth conditions (see text for details about the isolation of new strains) but show distinct different pattern of the BChl *c* homologs. Profiles were normalized to peak 2 for this comparison. Peaks were labeled according to Garcia Costas et al. (2012b) as shown in the inset table.

**FIGURE 13:**
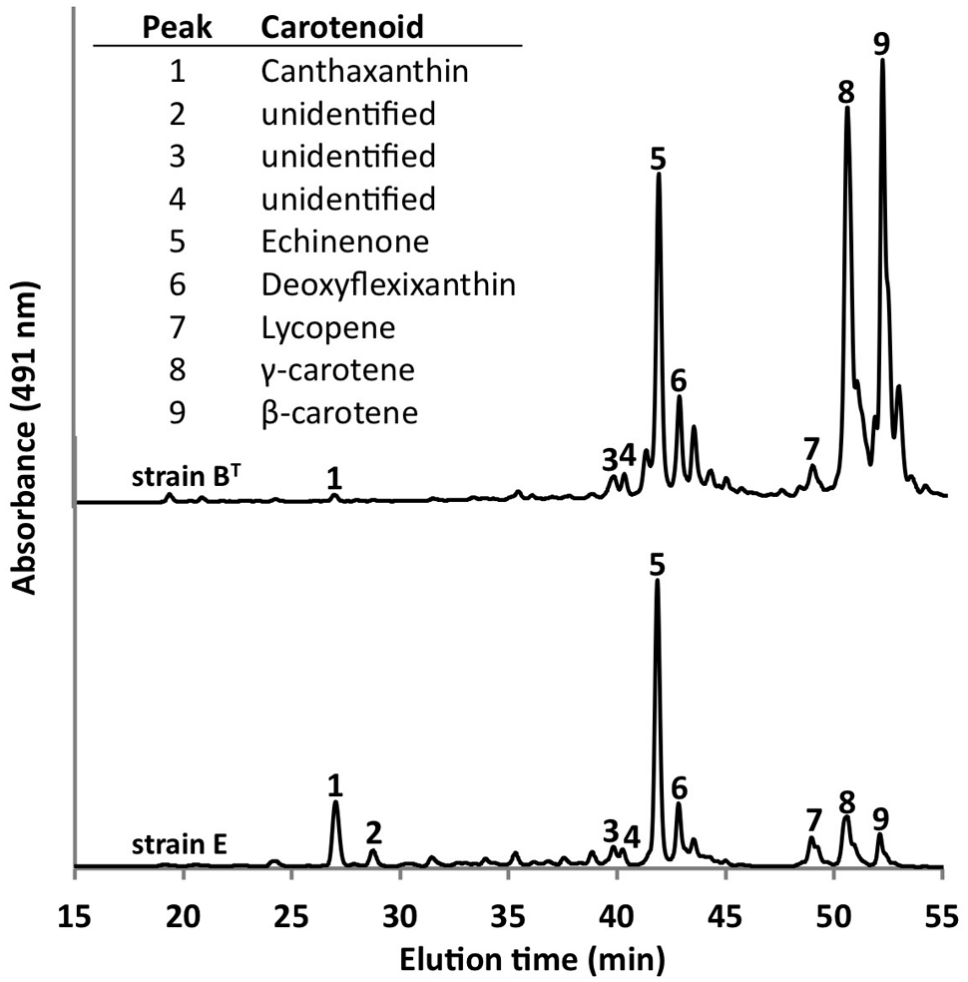
**Comparison of carotenoids in *C. thermophilum* strain B^**T**^ and strain E**. Both strains were cultivated under the same growth conditions (see text for details about the isolation of new strains), but they show differences in carotenoid content. The HPLC profile was normalized to peak 5 to facilitate the comparison. Peaks were labeled according to Garcia Costas et al. (2012b) as indicated in the inset table.

Using the optimized growth medium and growth conditions reported here, it was possible to grow new *C. thermophilum* strains and isolate representatives from mat samples taken at 52 and 60^∘^C samples in pure culture. In liquid medium the new strains form large cell aggregates and clumps, whereas strain B^T^ grows as a homogenous cell suspension (**Figure 11**). 16S rRNA sequence analyses showed that one new isolate is 100% identical to strain B^T^, whereas other representatives were only 99% identical in sequence to the lab strain B^T^ (data not shown). Pigment analyses and growth tests with strains only showing 99% similarity to the lab strain B^T^, respectively, also showed other differences from strain B^T^. Under the same growth conditions, CTM-medium 52^∘^C, pH 7.0, 50 μmol photons m^-2^ s^-1^ (from a tungsten source) and microoxic growth conditions, the new strains showed a distinctly different composition of BChl *c* homologs (**Figure 12**) and carotenoids (**Figure 13**) compared to the type strain B^T^. Consistent with the findings of Miller et al. (2009) and Garcia Costas (2010), initial experiments confirmed that different temperature ecotypes of *C. thermophilum* may exist in nature. The new strain can grow at temperatures as low as 39^∘^C whereas strain B^T^ is unable to grow at temperatures below 44^∘^C. Both strains have a similar growth optimum, ∼50^∘^C, and a similar upper limit of ∼58^∘^C under the conditions tested. In addition to ongoing growth and physiological testing, we plan to sequence the genome of at least one new strain and compare it with that of strain B^T^ in future studies. Besides the finding that the CTM-medium can be used for direct isolation of *C. thermophilum* from the environment, the new isolates will help us understand this unusual bacterium better.

### Conclusion

Axenic growth of *C. thermophilum* required the identification of essential nutrients on the one hand and the appropriate oxygen concentration on the other. The present study demonstrates that *C. thermophilum* is a microaerophilic, moderately thermophilic, anoxygenic, photoheterotrophic eubacterium. Its growth is absolutely dependent on several medium components, which include a reduced sulfur source, bicarbonate, all branched chain AAs, l-lysine and vitamin B_12_.

This study is an excellent example of how classical microbiology, in combination with modern –omics methods (Bryant et al., 2007; Liu et al., 2011, 2012; Garcia Costas et al., 2012a), led to the discovery and eventually to a fairly comprehensive characterization of this previously unknown bacterium. It was possible to develop a nutritionally defined medium to isolate *C. thermophilum* in axenic culture (Tank and Bryant, 2015). The growth conditions were further refined by determining the optimum pH, temperature and light intensities of *C. thermophilum*. The preceding –omics studies served as basis for developing specific hypotheses about the possible physiology of *C. thermophilum,* which were then tested with classical microbiology methods. This interplay of two different approaches led to the stepwise elimination of the co-culture contaminants, and in parallel we learned more about the physiology of *C. thermophilum*. Retrospectively, *C. thermophilum* is not a particularly extraordinary bacterium concerning its nutritional requirements and growth conditions. The major interest in *C. thermophilum* is due to it being the first chlorophototrophic member of the Acidobacteria. Furthermore, it shows characteristics that are usually either found in organisms that live under oxic or obligately anoxic conditions—combined into one organism. *C. thermophilum* uses AAs, peptides or proteins as a nearly universal supplier of major elements required for life. AAs can serve as carbon, nitrogen, sulfur and perhaps even energy source for *C. thermophilum*.

The ecological importance of *C. thermophilum* in the microbial mats it inhabits is still speculative, and this aspect was not a part of this study. According to previous metagenomic studies (Liu et al., 2011) and other molecular surveys (Miller et al., 2009), *C. thermophilum* represents only 5–10% of the chlorophototrophic mat communities in the alkaline hot spring mats it inhabits in Yellowstone National Park. *C. thermophilum* certainly benefits from other members of the mat community, because it relies on substrates produced by organisms in the mat, including AAs, reduced sulfur compounds, and CO_2_. In addition, *Synechococcus* spp. and *Roseiflexus* spp. are known to synthesize vitamin B_12_, which is essential for growth of *C. thermophilum* (Garcia Costas et al., 2011). On the other hand, *C. thermophilum* shows no biotin auxotrophy and could potentially provide this vitamin to the mat-dominant *Synechococcus* spp. for which biotin is essential. Future experiments will test this hypothesis by co-culturing *C. thermophilum* and *Synechococcous* sp. in a medium lacking biotin.

Now that *C. thermophilum* can easily be cultivated in axenic culture, it can be used in further studies to answer questions about the photosynthetic apparatus and about its ecological role in mats. This study nicely demonstrates that one can sometimes cultivate previously uncultivated organisms in axenic culture if one knows or can demonstrate the specific physiological and nutritional needs of the organism. It was clearly shown here that *C. thermophilum* has very simple nutrient requirements and that oxygen concentration played a crucially important role in its growth. The reason for the specific oxygen needs is the balance between oxygen-sensitive traits, for example, the type-1 photosynthetic reaction center, ferredoxin, 2-oxoglutarate ferredoxin oxidoreductase (Cabther_B0326), and possibly other enzymes containing iron-sulfur clusters on one hand, and components whose synthesis is oxygen-dependent, for example, Chl and BChls, oxygen-containing ketocarotenoids or tyrosine biosynthesis on the other.

An unexpected requirement for the cultivation of *C. thermophilum* was the need for bicarbontate, which possibly originates from the presence of CO_2_-incorporating enzymes, for example, the 2-oxoglutarate ferredoxin oxidoreductase (KDO, Cabther_B0326), phosphoenolpyruvate carboxylase (Cabther_A2240) or other enzymes involved in anaplerotic reactions. Heterotrophic CO_2_ fixation is also known to occur in aerobic anoxygenic phototrophic purple bacteria, which can produce up to 10% to 15% of their biomass from this process (Tang et al., 2009; Hauruseu and Koblížek, 2012). The contribution of bicarbonate/CO_2_ to the growth of *C. thermophilum* is not yet known, but it could be substantial. We hypothesize that succinyl-CoA is a major product of the degradation of branched chain AAs, and that this metabolite is then carboxylated by KDO to produce 2-oxoglutarate, which is a key precursor metabolite for the synthesis of proteins and (B)Chls. Because of the likely importance of this route for the production of metabolic precursors, it would be interesting to determine the oxygen sensitivity of KDO, which might partly explain the preference of this organism for microoxic conditions. The findings reported here concerning the oxygen relations of *C. thermophilum* suggest that oxygen concentration could prove to be a key factor in the isolation and cultivation of many other bacteria and archaea that have not yet been grown axenically.

## Conflict of Interest Statement

The authors declare that the research was conducted in the absence of any commercial or financial relationships that could be construed as a potential conflict of interest

## Abbreviations

BChl: bacteriochlorophyll
Chl: chlorophyll
HPLC: high performance liquid chromatography
